# A Case of Giant Cowper's Gland Syringocele in an Adult Male Patient

**DOI:** 10.1155/2015/682042

**Published:** 2015-08-30

**Authors:** Santosh Surana, Mohamed Elshazly, Adel Allam, Sateesh Jayappa, Deena AlRefai

**Affiliations:** ^1^Department of Radiology, Farwaniya Hospital, Ministry of Health, 92400 Kuwait, Kuwait; ^2^Department of Urology, Farwaniya Hospital, Ministry of Health, 92400 Kuwait, Kuwait

## Abstract

Cowper's gland syringocele is an uncommon, underdiagnosed cystic dilatation of Cowper's gland ducts showing various radiological patterns. Herein we report a rare case of giant Cowper's gland syringocele in an adult male patient, with description of MRI findings and management outcome.

## 1. Introduction

Cowper's gland syringocele is an uncommon, underdiagnosed cystic dilatation of Cowper's gland ducts showing various radiological patterns [1]. Syringoceles are traditionally known to affect the pediatric population but are rare in the adult population, presenting with urinary tract infection, gross hematuria, and voiding symptoms [2]. There are only 32 previous case reports of late presentation in adulthood [3]. Here, in this case report, we present a case of giant sized “infected Type-II perforated Cowper's gland syringocele” in an adult diagnosed initially by voiding cystourethrogram (VCUG) and later followed up by Magnetic Resonance Imaging (MRI).

## 2. Case Report

**Figure 1:**
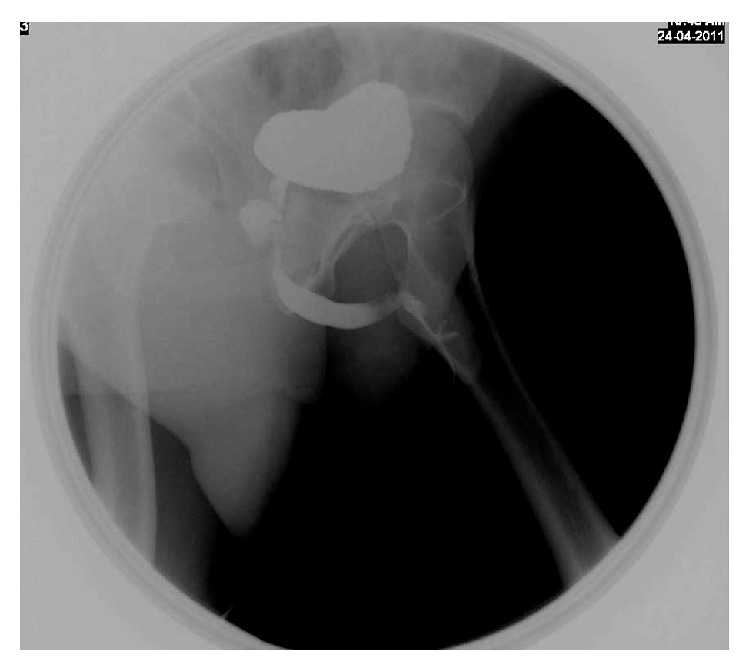
VCUG showing a small rounded cystic outpouching from the ventral aspect of the proximal bulbar urethra-Cowper's gland syringocele.

A 42-year-old male presented to emergency room (ER) with a large perineal swelling, fever, and dysuria. He had a history of acute urinary retention and perineal swelling 2 years ago. Emergency ultrasound at that time revealed a perineal cystic collection measuring 122 cc in volume with normal prostate and urinary bladder. Incision and drainage of the collection were performed. The content of the collection was serous in nature and proved to be urine on chemical analysis. VCUG study done 2 weeks later revealed a rounded cystic outpouching from the ventral aspect of the proximal bulbar urethra, measuring 2.5 × 2.5 × 2.3 cm in size (Figure 1). The diagnosis of Cowper's gland syringocele was suggested based on the anatomical location of Cowper's gland duct. Patient was discharged and advised for follow-up for further management. Unfortunately, patient missed follow-up for a long time of about 2 years of duration until he landed in the emergency department once again with the abovementioned symptoms.

**Figure 2:**
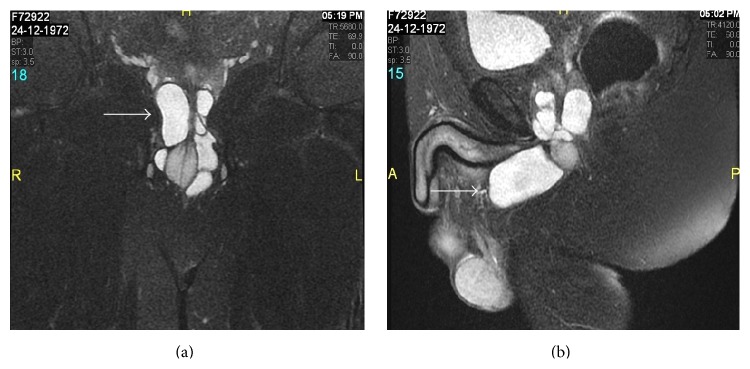
Coronal (a) and sagittal (b) T2-Fatsat images reveal hyperintense, multiloculated, tortuous, tubular, septated cystic structures encircling the compressed proximal bulbar urethra-giant sized Cowper's gland syringocele.

**Figure 3:**
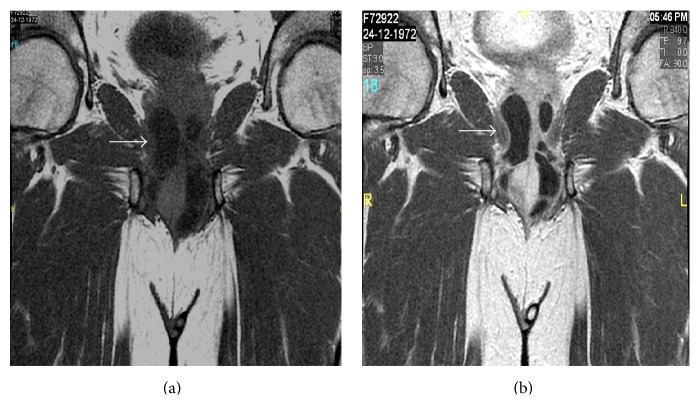
Coronal pre- (a) and post- (b) contrast T1WI images showing thick enhancing wall and septations of the cystic structures—infected giant sized Cowper's syringocele.

Pelvic MRI including urethra revealed large well-defined, multiloculated, tortuous, tubular, cystic structures measuring 7.5 × 3.0 × 2.7 cm in size, encircling the proximal bulbar urethra and extending up to the urogenital diaphragm, then coursing anteriorly, parallel to the ventral aspect of the distal bulbar urethra. Proximal bulbar urethra was also seen compressed by these cystic structures. The lesion appeared hyperintense on T2WI and T2-Fatsat (Figures 2(a) and 2(b)) and showed wall and septal enhancement on postgadolinium T1WI images (Figures 3(a) and 3(b)). The diagnosis of giant Cowper's gland syringocele with secondary infection was established, in view of the clinical details and previous VCUG findings. Patient was treated initially with antibiotics and later successful excision of the syringocele and repair of the urethra were performed through transperineal approach. Dissection of bulbous urethra with identification of Cowper's gland syringocele proximally to membranous urethra and distally to distal bulbar urethra was performed. Complete excision of the syringocele with closure of its connection to the urethra is performed. Postoperative follow-up was uneventful.

## 3. Discussion

Cowper's glands (bulbourethral glands) are a pair of pea-shaped exocrine glands in the male reproductive system that resides within the male urogenital diaphragm. Glands eventually form two collecting ducts, each measuring 2.5 cm in length. Although anatomic variations exist, the majority of ducts combine to make one confluent passage that opens at the posterior aspect of the bulbar urethra [4]. Secretions from these glands provide urethral lubrication and play an important role in the motility and protection of the sperms during ejaculation. Cowper's syringocele refers to the cystic dilatation of the ducts and is usually diagnosed in pediatric age group [5]. Syringoceles in adults are considered rare. However, a high index of suspicion can yield an increase in the detection of these lesions [6, 7].

The etiology is not clear. Both congenital and acquired types are described. Stasis and pressure changes may cause obstruction to the orifices of the bulbourethral duct resulting in accumulation of mucous and/or urine causing cystic dilatation. It may then lead to bacterial colonization and secondary infection [4]. Reviewing the literature, there are four reported types of syringocele described: Type-I: a simple syringocele with mild dilatation of the duct, Type-II: a perforated syringocele with dilated distal duct (downstream) that communicates with bulbar urethra via patulous ostium, Type-III: an imperforate syringocele that does not communicate with the urethra, and Type-IV: a ruptured syringocele. Functional and radiological Type-I, Type- II, and Type-IV are considered “open” lesions and are more likely to cause symptoms such as postvoid dribbling, purulent urethral discharge, and hematuria. Type-III lesions are considered as “closed” lesions and are more likely to cause obstructive symptoms, such as dysuria and urinary retention [8, 9].

The perforate syringocele can be easily seen with urethrography as a diverticulum communicating with the bulbar urethra as seen in our case. The imperforate syringocele is revealed indirectly as an eccentric mass impressing on the urethra usually with smooth margins. Syringocele can be confused with urethral diverticulum, partial urethral duplication, or an ectopic ureter and also needs to be differentiated from periurethral lesions such as abscesses (periurethral/perineal) and benign and malignant urethral tumors [4, 8].

Various noninvasive and invasive radiological procedures are used to diagnose Cowper's gland syringocele. Ultrasound sometimes visualizes “closed” cystic lesions in the anatomic region of Cowper's gland ducts. Urethrosonography is also used to diagnose “open” syringocele [2]. Retrograde and antegrade urethrogram/VCUG is a gold standard to confirm the diagnosis. Further work includes cystourethroscopy, urodynamic studies, computed tomography (CT), or Magnetic Resonance Imaging (MRI) [9].

MRI is a very useful noninvasive diagnostic modality having superior soft tissue resolution allowing a precise definition of the anatomic location, size, and extent of the cyst including delineation of the cyst wall, septae, and is contents [10]. It also helps in detection of complications such as secondary infection like in our case. In the literature, there is only a single case report of an adult imperforate Cowper's syringocele diagnosed by MRI [11]. Our case is the first report of a follow-up by MRI of a previously diagnosed small sized perforated syringocele by VCUG study, which had enlarged to a giant sized syringocele associated with secondary infection, and presented as a perineal abscess.

Asymptomatic syringoceles are often managed conservatively. Many symptomatic syringoceles eventually require surgical intervention [4]. In recent years, endoscopic unroofing of the cyst has been preferred in both open and closed types. Alternatively, open procedures such as transperineal ligation of Cowper's duct are performed, usually in cases of failed endoscopic unroofing. Open excision of the cyst and urethral repair are done, when a syringocele presents as a large perineal mass as in our case [12].

In summary, Cowper's gland syringocele should be included in the differential diagnosis of voiding dysfunction and perineal swelling in adults. MRI is very useful in delineating and characterizing the soft tissues due to its high resolution and reproducibility and in the differential diagnosis of Cowper's gland syringocele.
